# Anthrax in Albania: A Comprehensive Analysis of Epidemiology, Laboratory Diagnosis, and National Control Strategies in Animals

**DOI:** 10.3390/vetsci13030300

**Published:** 2026-03-22

**Authors:** Xhelil Koleci, Erson Dhimospira, Sulejman Kullolli, Mandy Elschner, Heinrich Neubauer, Gamal Wareth

**Affiliations:** 1 Department of Public Health, Veterinary Faculty of Tirana, Agricultural University of Tirana, Rr Pajsi Vodica, Koder-Kamez, 1029 Tirana, Albania; 2 National Agency of Veterinary and Plant Protection, 1001 Tirana, Albania; 3 Institute of Bacterial Infections and Zoonoses, Friedrich-Loeffler-Institut, 07743 Jena, Germany; 4 Department of Bacteriology, Immunology, and Mycology, Faculty of Veterinary Medicine, Benha University, Toukh 13736, Egypt; 5 Institute of Infectious Diseases and Infection Control, Jena University Hospital, 07747 Jena, Germany

**Keywords:** Albania, anthrax, vaccination, One Health, surveillance

## Abstract

Anthrax is one of the most significant zoonotic diseases in Albania due to its endemic presence in livestock, the potential for occupational exposure, and human cases. Although the implementation of risk-based livestock immunization, animal movement restrictions, and appropriate carcass disposal, the efficacy of targeted management remains limited in certain outbreaks due to insufficient enforcement of these measures. Their efficacy is specifically diminished by insufficient disinfection, the absence of grazing bans in contaminated pastures, and the absence of designated burial sites for the safe disposal of dead animals. District-level data on animal anthrax control programs were collected and analyzed for the period 2021–2025. In addition, a retrospective analysis of national datasets covering the same period was conducted using data from the national surveillance system, alongside a review of the relevant scientific and grey literature and aggregated program and routine surveillance data. Analysis showed that anthrax affected 149 animals in 97 farms, and the average number of animals per infected farm declined from 1.70 to 1.08, indicating a slight reduction within-farm outbreak. Hotspots for human anthrax were aligned with the animal cases and persisted particularly in the southern districts. The peak of outbreaks was in 2023, primarily driven by cattle (*n* = 32) and sheep (*n* = 24). Equine cases appeared only in 2024, with small clusters of 3 cases in both 2024 and 2025. Caprine cases remained consistently low throughout the period. Nevertheless, the number of outbreaks and within-herd cases are decreasing due to more rapid identification and response. Targeted surveillance on animal outbreaks provides critical insights into disease spread and links among affected farms in Albania. Therefore, One Health genomic surveillance and antibiotic susceptibility testing of *Bacillus anthracis* isolates are essential for understanding its epidemiology, transmission routes, and for tracing the sources of infection across humans, animals, and the environment.

## Simple Summary

Anthrax, caused by *Bacillus anthracis*, is a highly infectious disease that poses significant public health and veterinary health challenges. In Albania, it remains one of the leading zoonotic diseases, causing substantial problems in livestock populations. The current surveillance study analyzed national anthrax data from the past five years. It assessed the nationwide spatiotemporal distribution of cases and evaluated the strengths and weaknesses of the existing control program. The study provides detailed insights into ongoing anthrax outbreaks and evaluates laboratory diagnostic capacity and control measures implemented during this period. The results indicate that most cases and outbreaks are concentrated in the southern part of the country. Human cases show a similar geographic pattern, aligning with affected animal areas. Nevertheless, the overall number of outbreaks and within-herd cases has decreased, likely due to more rapid identification and improved response measures. Targeted surveillance of animal outbreaks provides critical insights into disease spread and epidemiological links among affected farms in Albania. Implementing a One Health approach is essential for a deeper understanding of the pathogen’s epidemiology and transmission routes. Such an approach would also enhance the ability to trace infection sources across humans, animals, and the environment.

## 1. Introduction

Anthrax, caused by *Bacillus* (*B.*) *anthracis*, is a significant threat to global health. It is a zoonotic infection that affects animal productivity and has serious implications for food safety, public health, and socioeconomic stability [1]. Anthrax is endemic in many regions worldwide, and climate change may further increase the risk of exposure for both humans and animals by affecting the persistence and spread of spores in the environment [2,3]. The Centers for Disease Control and Prevention (CDC) classified anthrax as one of the category A agents that can result in the greatest risk to national security [4,5,6]. *B. anthracis* is a Gram-positive, spore-forming bacterium typically found in soil and may be spread through wind, water, feed, and food [7]. Human anthrax infection occurs primarily through contact with infected animals or exposure to animal-derived materials contaminated with *B. anthracis* [8]. In herbivores like cattle and sheep, just a few hundred spores are enough for an infection; in humans, this number is significantly higher. Depending on the route of exposure, the disease may present as inhalational, cutaneous, gastrointestinal, or injection-associated anthrax. Cutaneous disease is the most frequently reported form, whereas inhalational and gastrointestinal anthrax are less common but often more severe if not treated promptly [8,9]. According to the anthrax annual epidemiological report 2021 published by the European Center for Disease Prevention and Control (ECDC), anthrax remains rare in humans across Europe but continues to occur sporadically, particularly in rural and agricultural areas with endemic animal infections with the highest numbers of registered cases among animals reported from Albania [10,11].

In Albania, anthrax remains a persistent zoonotic risk due to environmental contamination with spores and close interactions between humans and livestock in endemic areas. In 2011, Albania shifted from widespread, village-based vaccination to a risk-based control strategy. This approach emphasized immediate case reporting, rapid laboratory diagnosis, safe carcass disposal, and the targeted vaccination of animals at risk. The change was implemented following a period in which many animal deaths were reported as “suspected” anthrax cases without laboratory confirmation, with the aim of reducing environmental contamination and preventing new infections [7,12,13]. The policy adjustment was intended to reduce unnecessary vaccination and optimize resource allocation, but its epidemiological impact has not been fully evaluated [12]. Data from national surveillance programs between 2012 and 2018 indicate that both humans and animals continued to be affected. During this period, 142 laboratory-confirmed anthrax outbreaks in livestock were documented, suggesting localized but still persistent activity. A total of 242 human cases were recorded, with their geographical and temporal distribution largely mirroring that of animal outbreaks [12]. Animal anthrax is likely underreported, as some animal cases are only detected after associated human infections are identified, given the persistently high number of human cases relative to the number of laboratory-confirmed animal outbreaks [12,13]. The scientific literature has not adequately addressed the systematic assessment of temporal trends prior to and following the 2011 policy shift including epidemic intensity, spatial clustering, and recurrence patterns. To differentiate anthrax from other causes of sudden animal death and to direct public health interventions, accurate laboratory confirmation is crucial. Bacteriological culture, microscopy, and, when available, confirmatory molecular techniques are the main procedures used in Albanian laboratory diagnosis [12,13].

It is well-established that grazing on contaminated pastures, proximity to infected carcasses or contaminated sites, and the movement of animals in incubation increase the risk of animal infection. The incidence of outbreaks is influenced by a range of climatic and environmental factors, including alkaline or limestone-rich soils that promote spore persistence, dry conditions that encourage closer grazing, flooding or soil disturbance that exposes buried spores, and the accumulation of spores in low-lying areas following heavy rainfall [14,15]. Slaughtering sick animals, handling carcasses, and processing infected animal products (such as hides/skins and wool/hair) are the primary causes of human infection. Inadequate carcass disposal and environmental contamination associated with tanning activities also contribute to human risk. The national anthrax control strategy addresses these risks through rapid outbreak management measures including infectious source control, enforced restrictions on animal movement, mandatory suppressive immunization, safe disposal of animal carcasses, and effective disinfection procedures. This strategy is supported by an annually revised anthrax control program, trained emergency response teams, epidemiological investigations, and strengthened biosecurity measures. Although *B. anthracis* is genetically unique compared to many other bacterial diseases, molecular typing and whole-genome sequencing have demonstrated the existence of numerous evolutionary lineages with distinct geographic distributions [16,17,18]. There is currently a lack of information from areas of the Balkan region, and the genetic similarity of Albanian isolates to regional or global lineages is poorly documented. In endemic situations with persistent environmental reservoirs, genomic characterization can help distinguish between newly imported strains and recurrent re-emergence from historical foci, hence driving risk mapping and control strategies. Even though *B. anthracis* is still mostly susceptible to first-line antibiotics like doxycycline, ciprofloxacin, and penicillin, occasional reports of resistance determinants highlight the significance of routine surveillance. Antimicrobial susceptibility surveillance offers crucial assurance for treatment recommendations and planning in environments where outbreaks and empirical antibiotic use are prevalent. It is difficult to predict changes in pathogen features or assess possible changes in virulence or medication response patterns in the absence of structured genomic and susceptibility data [7,19,20].

Although Albania has implemented a national anthrax control program, its effectiveness is limited by the poor integration of surveillance data, weak involvement of local governments, and inadequate coordination between animal and human health sectors. These challenges undermine the successful application of a One Health approach.

In Ref. [13], to address this limitation, we compiled and analyzed anthrax surveillance and laboratory data collected between 2021 and 2025. This study aimed to assess temporal trends in outbreak intensity, identify geographic clustering, and characterize recent incidence patterns. The findings are intended to support a One Health-based, risk-oriented strategy for vaccination, the regulation of livestock movement, and improved management of infected carcasses.

## 2. Materials and Methods

In the current study, we conducted a retrospective descriptive review of anthrax in livestock, one of Albania’s most important zoonotic bacterial diseases. Data on livestock anthrax, including the number of laboratory-confirmed cases, the affected animal species, the district of occurrence, the date of outbreak confirmation, and vaccination data were provided by the national veterinary authority. Any sudden death with symptoms consistent with anthrax and a positive screening test based on the microscopic identification of *B. anthracis* in stained smears was considered a suspected anthrax case. Only after *B. anthracis* was isolated in a bacteriological culture at the national reference laboratory was a confirmed case reported. Based on laboratory-confirmed cases recorded over the last three to five years, the number of vaccinated animals represents all animals classified as at risk and vaccinated. The yearly totals of animal cases were calculated, subsequently, we computed each district’s proportionate part of the national case burden, the five-year cumulative incidence per district, and the absolute and percentage year-over-year increases. Geographic clustering was assessed descriptively by identifying districts that made a disproportionate contribution to cumulative incidence. Regular surveillance records for livestock anthrax were analyzed to describe temporal and spatial patterns of disease occurrence. After the data were imported into “*R*” software, standardized variables were added to each dataset, including year or period, grouping variables (species, district, or data source), and case counts (restricted to non-negative integers).

To guide the narrative synthesis and support hotspot identification, we calculated the annual case totals, absolute and percentage year-over-year (YoY) changes, and five-year cumulative totals by species and district, along with their proportional contributions. In addition, we identified “top cells”, defined as district–year or species–year combinations with the highest reported case counts, to highlight areas and periods of intensified transmission.

We employed a Monte Carlo simulation framework in R (version 4.2) to quantify forecast uncertainty for future national totals and district–year case counts. Poisson regression or negative binomial regression models were used depending on the degree of overdispersion in the data. Predictive distributions were generated by simulating from the fitted model parameters and sampling expected counts to derive uncertainty intervals.

To evaluate the robustness of the forecasts, we conducted sensitivity analyses that examined:

1. Different forecast intervals,
2. The inclusion of a quadratic time term (poly (Year, 2)),
3. Adjustments for variations in the population at risk (incorporating an offset when data were available), and
4. The impact of removing year-to-date (YTD) and combined reporting periods.

Data management, analysis, and visualization were performed in R (version 4.2) using the tidyverse packages (dplyr, tidyr, ggplot2, forcats), as well as MASS, broom, viridis, and conflicted.

## 3. Results

**Figure 1:**
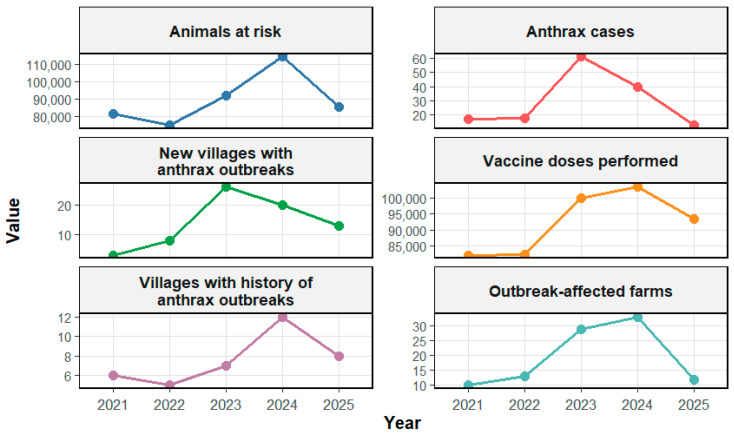
Trends in anthrax-related surveillance and control indicators from 2021 to 2025, such as outbreak-affected farms, animals at risk, vaccination doses administered, villages with a history of outbreaks, and new outbreak villages.

**Figure 2:**
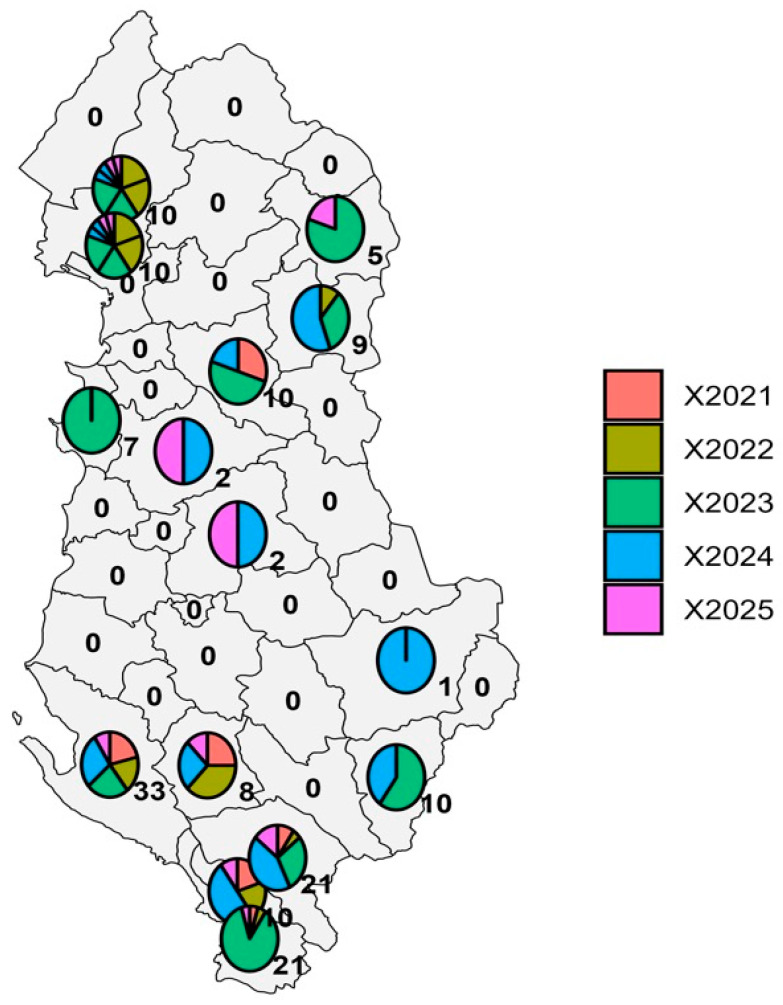
Anthrax cases by district and year in Albania (2021–2025). The map displays the number of cases by year (2021–2025) and district. According to the pie charts’ color composition, 2023 (green) produced the greatest percentage of cases in numerous districts, which is consistent with the year’s nationwide peak. On the other hand, 2025 (purple) showed fewer cases, which reflects the drop in outbreaks that were recorded in the study’s last year. Overall, the map shows ongoing endemic activity in some southern areas, but from 2021 to 2025, outbreaks were either intermittent or nonexistent throughout most of the nation.

**Figure 3:**
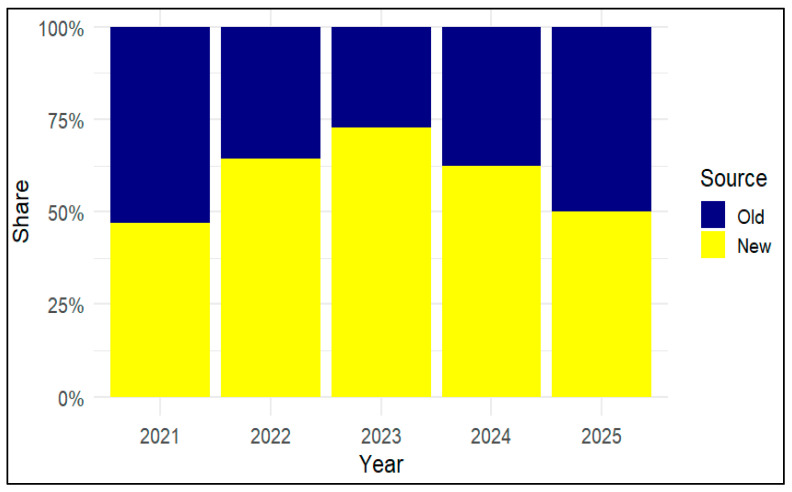
Proportion of new versus previously affected anthrax villages by year (2021–2025). The figure shows that new sources of infection dominated in most years, especially in 2023, when they accounted for almost three-quarters of all sources. Old sources were more important in 2021 than they were in decline, and by 2025, the two types contributed roughly equally. The source refers to the origin or reservoir where the infection likely came from.

**Figure 4:**
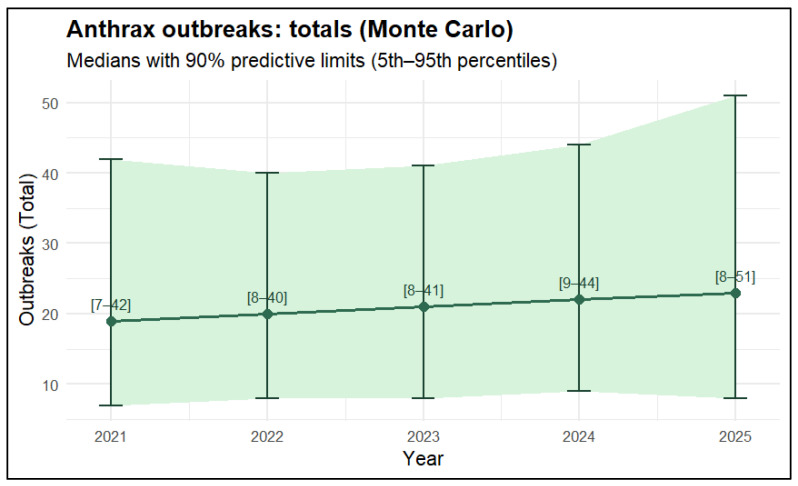
Anthrax outbreaks: Totals with 90% predictive limits (Monte Carlo, 2021–2025). The median predicted number of outbreaks from 2021 to 2025 was slightly higher according to the models, although the 90% prediction intervals were broad and mostly overlapped between years. The very wide range for 2025 limits the deductive power of any apparent change and shows significant uncertainty, especially in the upper range of the potential outbreak totals.

**Figure 5:**
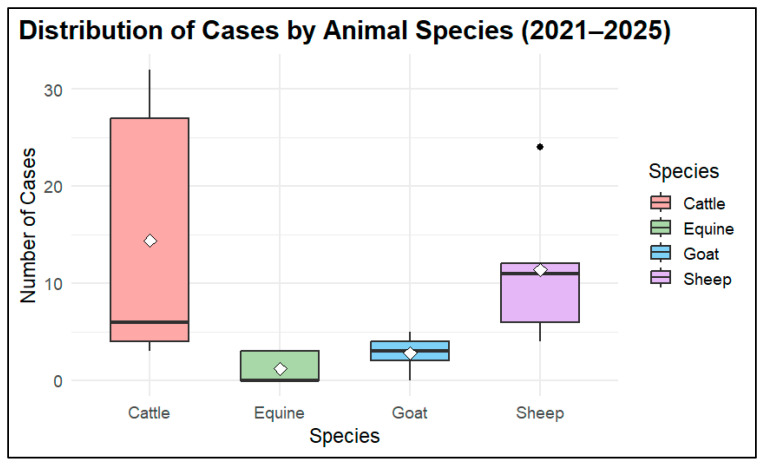
Anthrax cases by animal species (2021–2025). The boxplot shows the distribution of annual anthrax cases reported in each animal species from 2021 to 2025. Each box represents the spread of values for that species, including the median, interquartile range, and any outliers.

The findings from the analysis of anthrax outbreaks during the 2021–2025 study period are summarized in Table 1 and Table 2 and Figure 1, Figure 2, Figure 3, Figure 4 and Figure 5. These visualizations provide an overview of anthrax trends in Albanian livestock over five years, capturing temporal dynamics, spatial distribution, and key outbreak characteristics.

The dataset indicates an expansion in the geographic extent of affected areas and an increase in the population of at-risk animals (defined as susceptible animals located within epidemiological units that reported at least one confirmed anthrax case in the previous five years) particularly during 2023–2024. This period also coincided with higher immunization coverage, which appears to have contributed to a reduction in reported case numbers in 2025.

Overall, the integrated tables and figures offer a multi-year perspective on anthrax epidemiology, enabling comparisons across species, districts, and time periods. These insights are critical for informing risk-based allocation of resources, guiding targeted vaccination campaigns, and supporting decision-making within Albania’s One Health surveillance framework. By linking temporal trends, spatial hotspots, and immunization data, this analysis provides a robust evidence base for strategic planning and outbreak prevention.

Anthrax surveillance data from 2021 to 2025 recorded 149 affected animals across 97 positive farms, indicating that outbreaks, while occasional, persisted throughout the study period (Table 1). The average number of affected animals per positive farm declined from 1.70 in 2021 to 1.08 in 2025, suggesting more effective herd confinement. This reduction in outbreak size likely reflects improved field response, early detection, and strengthened biosecurity in high-risk areas, although annual fluctuations obscure the overall pattern. The observed downward trend, however, was not statistically significant. The data showed a peak in 2023, primarily driven by cattle (*n* = 32) and sheep (*n* = 24). Cattle cases then declined to 27 in 2024 and 6 in 2025. Following a sharp rise in ovine cases in 2023, numbers decreased over 2024–2025. Equine cases appeared only in 2024, with small clusters of 3 cases in both 2024 and 2025. Caprine cases remained consistently low throughout the period (Table 2).

Although vaccination coverage increased, outbreaks continued to occur in newly affected areas, suggesting that broader vaccination alone did not consistently prevent spread and was not always well-matched to where risk emerged. Under the national anthrax control strategy, preventive vaccination is targeted to villages/epidemiological units with a recent history of animal anthrax—previously defined as the last five years, and more recently applied for three years following an outbreak

Several factors may have contributed to variations in the anthrax epidemic activity from 2021 to 2025. From 2023–2024, the number of animals susceptible to anthrax within epidemiological units that had reported at least one confirmed case in the previous five years (“animals at risk”) increased. The epidemiological unit is generally defined at the level of a village or administrative unit. This is because animals from different farms or owners often share common pastures, water sources, and paths or roads, creating frequent contact between herds and increasing the risk of disease transmission. This increase was accompanied by a significant rise in vaccination doses, reflecting strengthened preventive measures in high-risk areas, which likely contributed to the decline in both the number of new affected villages and anthrax cases from 2024 to 2025 (Figure 1). The rise in “animals at risk” is due to the expansion of epidemiological units in the context of recently identified outbreaks. In these regions, vaccination is administered reactively in accordance with national protocol, focusing on animals that are part of the designated outbreak unit. Therefore, rather than being an independent biological measure of risk, this indicator is administrative and depends on epidemic reporting.

The spatiotemporal analysis of anthrax cases shows that 14 out of Albania’s 36 districts have documented outbreaks, with clear geographic clustering. The heatmap in Figure 2 indicates a significant increase in 2023, driven by spikes in Durrës (7 cases), Kolonjë (6 cases), Gjirokastër (6 cases), and Mat (5 cases), with the highest concentration in Sarandë (18 cases). Activity remained high in parts of the south during 2024, with Gjirokastër peaking again at 9 cases and Vlorë maintaining consistently high levels over five years (7, 6, 8, 9, 3 cases). Delvinë also saw an increase in 2024, with 5 cases before declining. In contrast, several districts such as Tiranë, Elbasan, and Korçë reported only isolated or sporadic cases, mostly late in the series. Overall, anthrax activity stayed locally elevated in the southern and coastal regions (Vlorë, Sarandë, Gjirokastër, Delvinë), with a general decline noted in the first five months of 2025. This geographical persistence suggests that these hotspots need enhanced surveillance, risk-based vaccination prioritization of animals and areas with the highest likelihood of exposure, and targeted control measures (Figure 2).

Stacked proportionate bars (Figure 3) illustrate the distribution of anthrax outbreaks between newly affected villages (“New”, villages had no previously recorded laboratory-confirmed anthrax cases) and historically affected villages (“Old” villages had confirmed cases recorded previously (recurrent locations)) from 2021 to 2025. In 2021, both sources contributed roughly equally. The proportion of newly affected villages increased sharply in 2022, peaking in 2023, indicating geographic expansion. In 2024, outbreaks shifted back to previously affected areas, while early 2025 was dominated by old-source cases, suggesting reactivation or re-exposure in known hotspots. These trends reflect alternating phases of emergence, with widespread regional expansion in 2023 followed by consolidation in 2024–2025.

The Monte Carlo explorations suggest a small increase in the median number of anthrax outbreaks from 2021 to 2025 (approximately 19 to 23), but the 90% predictive intervals were wide and largely overlapping across years. This pattern is consistent with a broadly stable outlook under the observed reporting conditions rather than evidence of a systematic rise. In Figure 4, the green ribbon and brackets represent the 5th to 95th percentiles of the simulated outbreak totals.

The distribution of anthrax cases over five years across four animal species: cattle, sheep, goats, and horses, are shown in Figure 5. Cattle were most affected by anthrax outbreaks (*n* = 72 cases), as indicated by the highest variability and median number of cases. In sheep, there were moderate case numbers (*n* = 57) with noticeable annual fluctuations, while goats and horses showed consistently low case counts (14 in goats and 6 in equine), with equine cases appearing only in recent years. Overall, the plot suggests that cattle were the most vulnerable hosts or were most often exposed to the primary source of infection during the studied period (Figure 5).

## 4. Discussion

Anthrax is a zoonotic disease caused by a spore-forming bacterium that can persist in soil for decades. In humans, the most common presentation is cutaneous anthrax, whereas inhalational and gastrointestinal forms are less frequent but considerably more severe [5,6]. Given that transmission occurs at the interface of animals, humans, and the environment, anthrax exemplifies a disease well-suited to the One Health approach.

Anthrax has historically been present in Albania, particularly as a zoonotic disease affecting livestock and occasionally humans [17]. The disease remains endemic in various regions of the country, particularly in the southern areas, including Gjirokastër, Tepelenë, Sarandë, and Delvinë, where environmental factors such as calcium-rich soils may enhance the long-term persistence of spores in the environment [19]. In the past decade, Albania has reported a comparatively high number of livestock anthrax cases in comparison to other European countries, with one study documenting approximately 28 cases in domestic animals over a multi-year period [3,10]. Previous genotyping studies have shown that *B. anthracis* isolates recovered from soil and animal carcasses predominantly belong to closely related Trans-European lineage A.Br.008/009 genotypes (Albania GT/1–GT/3) [19,20]. These findings support the hypothesis of a long established local ancestral strain that intermittently causes outbreaks.

The primary method of anthrax surveillance is passive reporting, which means that cases are found when farmers or veterinarians report unexpected mortality or suspected disease. Although veterinary services have a comparatively high level of awareness, underreporting is probably common, especially when it comes to animals. There is evidence of incomplete carcass disposal, delayed notification, and illegal or emergency slaughter of infected animals, all of which can prevent cases from being reported to the official surveillance system. Human anthrax cases are still somewhat common and frequently arise after animal exposure, which strongly implies that some animal cases are overlooked or only reported after human infection [12].

Albania’s current anthrax control program is centered on risk-based vaccination, a targeted strategy in which vaccines are administered to animals in regions with the highest likelihood of exposure, rather than vaccinating all animals indiscriminately. The program also includes the laboratory confirmation of cases by pathogen isolation, mandatory reporting of suspected infections, and passive surveillance. Surveillance relies on notifications from farmers and official veterinarians, which are followed by field investigations, proper sample collection (blood and swabs, no organs), and laboratory diagnostic testing. Preventive vaccination is administered for three years after an outbreak, targeting villages or epidemiological units with a recent history of anthrax. During active outbreaks, suppressive vaccination is implemented at affected sites. The program also emphasizes rapid response, enforcement of animal movement restrictions, proper carcass disposal, and environmental disinfection using high-concentration formaldehyde or glutaraldehyde solutions in accordance with national guidelines to minimize environmental contamination and prevent further spread [12]. The village is commonly used as the epidemiological unit, as sheep, goats, and cattle often graze together and share the same routes, paths, and water sources. Preventive vaccination targets villages with a recent history of anthrax (previously five years and currently three years following an outbreak), while suppressive vaccination is compulsory during outbreaks. When a case is confirmed in culture isolations, all susceptible animals within the affected village/epidemiological unit are vaccinated with a certified vaccine administered subcutaneously by official veterinary services [12,13].

The active anthrax control program implemented across Albanian districts from 2021 to 2025 demonstrates stable spatial patterns but also time-dependent fluctuations that are informative for disease control strategies, despite inherent biological variability. Cattle and sheep cases emerged as the primary contributors to the pronounced temporal increase in numbers observed in 2023 (*n* = 61), followed by a decline in 2024 (*n* = 40), and low year-to-date (YTD) incidence in 2025 (*n* = 13). The source (origin or reservoir of the infection or outbreak) attribution analyses for the period 2022–2024 indicate a temporary expansion into previously unaffected areas, whereas data from 2025 suggest a revival of established endemic foci. Spatially, reported cases were concentrated along the southern and coastal corridor encompassing Vlorë, Sarandë, Gjirokastër, and Delvinë in spring and summer, aligning with seasonal livestock movements to historically contaminated grazing lands and the persistence of spores. Outbreaks in newly affected regions may be driven by factors beyond vaccination coverage. Although preventive vaccination is implemented according to the national strategy and targeted to villages or epidemiological units with a recent history of anthrax (previously five years, now three years post-outbreak), disease emergence in new areas suggests that risk changes over time. Factors such as uncontrolled animal movements, shared pastures and watering points, and environmental contamination combined with meteorological and climatic events (e.g., flooding followed by prolonged hot and dry periods) may increase the exposure to spores [7,18]. However, in the absence of field-based epidemiological studies linking outbreaks to specific transmission pathways, these explanations remain largely inferential. Although variations in reporting cannot be excluded, the subsequent decline in incidence likely reflects the impact of targeted interventions, including enhanced surveillance and catch-up vaccination campaigns [12,20].

Ongoing priorities include implementing ring vaccination zones around affected herds, ensuring strict adherence to safe carcass disposal practices, and sustaining pre-season immunization in areas with recurrent outbreaks. In light of the spread of anthrax into new areas during 2022–2024, surveillance efforts should be strengthened by rapid outreach to smallholder farmers and risk-based environmental assessments i.e., mapping of historical carcass burial sites and monitoring soil disturbance [12,20].

In this study, uncertainty was clearly assessed to avoid overinterpreting point estimates. Because of this, the results are displayed as expected medians with 90% uncertainty intervals generated from Monte Carlo simulation in situations when data are limited and subject to change, reflecting both process variability and parameter uncertainty.

## 5. Limitations of the Study

This analysis was limited by the low annual case counts and short five-year time series, which impair statistical power and make it more prone to random fluctuation. Passive surveillance data, which are susceptible to underreporting, inconsistent laboratory confirmation, and possible shifts in detection or reporting over time, were used in the research. For irregular reporting intervals, such as year-to-date reporting for 2025, data constraints necessitated approximation. Strong assessment of control efficacy was hindered by the lack of denominator-based vaccination coverage, herd structure, animal movement data, environmental exposure indicators, and operational response metrics. These restrictions further limited predictive modeling; broad and overlapping uncertainty intervals indicate assumptions that may reflect reporting artifacts as much as epidemiological dynamics and should be considered exploratory uncertainty quantification rather than accurate forecasting. Finally, it is difficult to determine whether observed “new” outbreaks reflect regional extension or the reactivation of pre-existing environmental reservoirs due to the lack of contemporaneous genetic data.

## 6. Conclusions

Between 2021 and 2025, anthrax outbreaks continued in Albania, highlighting the necessity of ongoing prevention and response strategies, including vaccination, when necessary, safe carcass care, and surveillance that records both repeated foci and recently discovered locations. Integrated datasets include testing intensity, denominator-based vaccination coverage, and animal movement data will be necessary for a more thorough assessment of intervention efficacy. It continues to be important to strengthen One Health coordination, especially through the genetic characterization of circulating isolates, testing for antibiotic susceptibility, and the rigorous study of potential sources of infection (including contamination sites). Clearer tracing of transmission pathways and more evidence-based targeting of control and prevention efforts would be made possible by connecting these data to field epidemiology.

## Figures and Tables

**Table 1:** Integrated surveillance indicators for anthrax in livestock in Albania, 2021–2025.

Indicator	2021	2022	2023	2024	2025	Total
Number of villages with a history of anthrax outbreaks	6	5	7	12	8	38
Number of new villages with anthrax outbreaks	3	8	26	20	13	70
Number of animals at risk	81,800	75,333	91,953	114,143	85,384	448,613
Vaccine doses performed	82,189	82,300	99,901	103,366	93,522	461,278
Anthrax cases	17	18	61	40	13	149
Outbreak-affected farms (count)	10	13	29	33	12	97

**Table 2:** Number of confirmed cases of anthrax in animals by species, 2021–2025.

Year	Equine	Cattle	Sheep	Goat	Total
2021	0	3	11	3	17
2022	0	4	12	2	18
2023	0	32	24	5	61
2024	3	27	6	4	40
2025	3	6	4	0	13
Total	6	72	57	14	149

## Data Availability

The original contributions presented in this study are included in the article. Further inquiries can be directed to the corresponding authors.

## Abbreviations

The following abbreviations are used in this manuscript:

*B. anthracis*	*Bacillus anthracis*
CDC	The Centers for Disease Control and Prevention
YoY	Year-over-year
